# Supplementary material for: Enhancing Cellular Uptake of Native Proteins through Bio-Orthogonal Conjugation with Chemically Synthesized Cell-Penetrating Peptides
Source: Pharmaceutics. 2024 May 3;16(5):617. doi: 10.3390/pharmaceutics16050617 (PMC11125112; doi:10.3390/pharmaceutics16050617)
Supplement: Supplementary file 1 [file pharmaceutics-16-00617-s001.zip › pharmaceutics-2973100-supplementary.pdf]

SUPPLEMENTARY INFORMATION

Figure S1. Plasmids used in this work.

Name	Backbone	Insert sequence
p_mCherry_EGFPn_inteinN	pEF1α-mCherry-C1 (Clontech, Catalog No. 631972)	ATGGTGAGCAAGGGCGAGGAGGATAACATGGCCATCATCAAGGAGTTCATGCGCT<br>TCAAGGTGCACATGGAGGGCTCCGTGAACGGCCACGAGTTCGAGATCGAGGGCGA<br>GGGCGAGGGCCGCCCCTACGAGGGCACCCAGACCGCCAAGCTGAAGGTGACCAA<br>GGGTGGCCCCCTGCCCTTCGCCTGGGACATCCTGTCCCCTCAGTTCATGTACGGCTC<br>CAAGGCCTACGTGAAGCACCCCGCCGACATCCCCGACTACTTGAAGCTGTCCTTCC<br>CCGAGGGCTTCAAGTGGGAGCGCGTGATGAACTTCGAGGACGGCGGCGTGGTGAC<br>CGTGACCCAGGACTCCTCCCTGCAGGACGGCGAGTTCATCTACAAGGTGAAGCTGC<br>GCGGCACCAACTTCCCCTCCGACGGCCCCGTAATGCAGAAGAAGACCATGGGCTG<br>GGAGGCCTCCTCCGAGCGGATGTACCCCGAGGACGGCGCCCTGAAGGGCGAGATC<br>AAGCAGAGGCTGAAGCTGAAGGACGGCGGCCACTACGACGCTGAGGTCAAGACC<br>ACCTACAAGGCCAAGAAGCCCGTGCAGCTGCCCGGCGCCTACAACGTCAACATCA<br>AGTTGGACATCACCTCCCACAACGAGGACTACACCATCGTGGAACAGTACGAACG<br>CGCCGAGGGCCGCCACTCCACCGGCGGCATGGACGAGCTGTACAAGTCCGGAGCG<br>GGCAGCGCGGCGGGCAGCGGCGAATTTCCTGGCAGCATGAGCAAAGGAGAAGAA<br>CTGTTTACCGGAGTGGTGCCAATTCTCGTGGAACTGGATGGCGATGTTAATGGGCA<br>CAAATTTTCTGTCAGAGGAGAAGGTGAAGGTGATGCCACAATTGGAAAGCTCACT<br>CTGAAATTCATCTGCACCACTGGAAAACTCCCTGTGCCATGGCCAACACTGGTCAC<br>TACCTTAACCTACGGCGTGCAGTGCTTTTCAAGATATCCAGATCATATGAAAAGGC<br>ATGACTTTTTCAAATCAGCAATGCCCGAGGGGTATGTGCAGGAGCGAACGATTTCT<br>TTCAAAGATGATGGGAAATACAAGACCCGCGCTGTTGTTAAGTTTGAAGGAGACG<br>ATAAAGGCATTGAATGCAGCGTGTTTAGCATTGATAGCAACGGCATTGTGTATACC<br>CAGCCGATTGCGCAGTGGCATCATCGCGGCAAACAGGAAGTGTTTGAATATTGCCT<br>GGAAGATGGCAGCATTATTAAAGCGACCAAAGATCATAAATTTATGACCCAGGAT<br>GGCAAAATGCTGCCGATTGATGAAATTTTTGAACAGGAACTGGATCTGCTGCAGGT<br>GAAAGGCCTGCCGGAATAAG
p_inteinC_EGFPC_SBFP	pCMV-GFP (Addgene #11153)	ATGGTGAGCAAGGGCGAGGAGGATAACATGGCCATCATCAAGGAGTTCATGCGCT<br>TCAAGGTGCACATGGAGGGCTCCGTGAACGGCCACGAGTTCGAGATCGAGGGCGA<br>GGGCGAGGGCCGCCCCTACGAGGGCACCCAGACCGCCAAGCTGAAGGTGACCAA<br>GGGTGGCCCCCTGCCCTTCGCCTGGGACATCCTGTCCCCTCAGTTCATGTACGGCTC<br>CAAGGCCTACGTGAAGCACCCCGCCGACATCCCCGACTACTTGAAGCTGTCCTTCC<br>CCGAGGGCTTCAAGTGGGAGCGCGTGATGAACTTCGAGGACGGCGGCGTGGTGAC<br>CGTGACCCAGGACTCCTCCCTGCAGGACGGCGAGTTCATCTACAAGGTGAAGCTGC<br>GCGGCACCAACTTCCCCTCCGACGGCCCCGTAATGCAGAAGAAGACCATGGGCTG<br>GGAGGCCTCCTCCGAGCGGATGTACCCCGAGGACGGCGCCCTGAAGGGCGAGATC<br>AAGCAGAGGCTGAAGCTGAAGGACGGCGGCCACTACGACGCTGAGGTCAAGACC<br>ACCTACAAGGCCAAGAAGCCCGTGCAGCTGCCCGGCGCCTACAACGTCAACATCA<br>AGTTGGACATCACCTCCCACAACGAGGACTACACCATCGTGGAACAGTACGAACG<br>CGCCGAGGGCCGCCACTCCACCGGCGGCATGGACGAGCTGTACAAGTCCGGAGCG<br>GGCAGCGCGGCGGGCAGCGGCGAATTTCCTGGCAGCATGAGCAAAGGAGAAGAA<br>CTGTTTACCGGAGTGGTGCCAATTCTCGTGGAACTGGATGGCGATGTTAATGGGCA<br>CAAATTTTCTGTCAGAGGAGAAGGTGAAGGTGATGCCACAATTGGAAAGCTCACT<br>CTGAAATTCATCTGCACCACTGGAAAACTCCCTGTGCCATGGCCAACACTGGTCAC<br>TACCTTAACCTACGGCGTGCAGTGCTTTTCAAGATATCCAGATCATATGAAAAGGC<br>ATGACTTTTTCAAATCAGCAATGCCCGAGGGGTATGTGCAGGAGCGAACGATTTCT<br>TTCAAAGATGATGGGAAATACAAGACCCGCGCTGTTGTTAAGTTTGAAGGAGACG

		ATAAAGGCATTGAATGCAGCGTGTTTAGCATTGATAGCAACGGCATTGTGTATACC<br>CAGCCGATTGCGCAGTGGCATCATCGCGGCAAACAGGAAGTGTTTGAATATTGCCT<br>GGAAGATGGCAGCATTATTAAAGCGACCAAAGATCATAAATTTATGACCCAGGAT<br>GGCAAAATGCTGCCGATTGATGAAATTTTTGAACAGGAACTGGATCTGCTGCAGGT<br>GAAAGGCCTGCCGGAATAAG
p_mCherry_EGFP1-10_inteinN	pEF1α-mCherry-C1 (Clontech, Catalog No. 631972)	ATGGTGAGCAAGGGCGAGGAGGATAACATGGCCATCATCAAGGAGTTCATGCGCT<br>TCAAGGTGCACATGGAGGGCTCCGTGAACGGCCACGAGTTCGAGATCGAGGGCGA<br>GGGCGAGGGCCGCCCCTACGAGGGCACCCAGACCGCCAAGCTGAAGGTGACCAA<br>GGGTGGCCCCCTGCCCTTCGCCTGGGACATCCTGTCCCCTCAGTTCATGTACGGCTC<br>CAAGGCCTACGTGAAGCACCCCGCCGACATCCCCGACTACTTGAAGCTGTCCTTCC<br>CCGAGGGCTTCAAGTGGGAGCGCGTGATGAACTTCGAGGACGGCGGCGTGGTGAC<br>CGTGACCCAGGACTCCTCCCTGCAGGACGGCGAGTTCATCTACAAGGTGAAGCTGC<br>GCGGCACCAACTTCCCCTCCGACGGCCCCGTAATGCAGAAGAAGACCATGGGCTG<br>GGAGGCCTCCTCCGAGCGGATGTACCCCGAGGACGGCGCCCTGAAGGGCGAGATC<br>AAGCAGAGGCTGAAGCTGAAGGACGGCGGCCACTACGACGCTGAGGTCAAGACC<br>ACCTACAAGGCCAAGAAGCCCGTGCAGCTGCCCGGCGCCTACAACGTCAACATCA<br>AGTTGGACATCACCTCCCACAACGAGGACTACACCATCGTGGAACAGTACGAACG<br>CGCCGAGGGCCGCCACTCCACCGGCGGCATGGACGAGCTGTACAAGTCCGGAGCG<br>GGCAGCGCGGCGGGCAGCGGCGAATTTCCTGGCAGCATGAGCAAAGGAGAAGAA<br>CTGTTTACCGGAGTGGTGCCAATTCTCGTGGAACTGGATGGCGATGTTAATGGGCA<br>CAAATTTTCTGTCAGAGGAGAAGGTGAAGGTGATGCCACAATTGGAAAGCTCACT<br>CTGAAATTCATCTGCACCACTGGAAAACTCCCTGTGCCATGGCCAACACTGGTCAC<br>TACCTTAACCTACGGCGTGCAGTGCTTTTCAAGATATCCAGATCATATGAAAAGGC<br>ATGACTTTTTCAAATCAGCAATGCCCGAGGGGTATGTGCAGGAGCGAACGATTTCT<br>TTCAAAGATGATGGGAAATACAAGACCCGCGCTGTTGTTAAGTTTGAAGGAGACA<br>CACTGGTGAATCGTATCGAGTTGAAGGGGACAGACTTTAAGGAAGACGGAAATAT<br>TCTCGGGCACAAGCTGGAATACAACTTTAATAGTCACAATGTGTATATCACAGCGG<br>ACAAGCAAAAGAATGGTATCAAAGCAAACTTCACAGTCAGACACAACGTCGAAG<br>ATGGAAGCGTTCAGCTGGCCGACCATTATCAACAGAACACTCCAATCGGGGACGG<br>CCCTGTGCTCCTCCCAGACAACCATTACCTGTCAACCCAGACTGTCCTAAGTAAAG<br>ATCCAAATGAAAAAGATAAAGGCATTGAATGCAGCGTGTTTAGCATTGATAGCAA<br>CGGCATTGTGTATACCCAGCCGATTGCGCAGTGGCATCATCGCGGCAAACAGGAA<br>GTGTTTGAATATTGCCTGGAAGATGGCAGCATTATTAAAGCGACCAAAGATCATAA<br>ATTTATGACCCAGGATGGCAAAATGCTGCCGATTGATGAAATTTTTGAACAGGAAC<br>TGGATCTGCTGCAGGTGAAAGGCCTGCCGGAATAAG
p_inteinC_EGFP11_SBFP	pCMV-GFP (Addgene #11153)	ATGATTAAAATTGCGACCCGCAAATATCTGGGCAAACAGAACGTGTATGATATTGG<br>CGTGGAACGCGATCATAACTTTGCGCTGAAAAACGGCTTTATTGCGAGCAACTGCT<br>TTAACAAACGGGACCACATGGTGCTGCACGAGTACGTGAACGCCGCCGGCATCAC<br>AGGCAGCGCGGGCAGCGCGGCGGGCAGCGGCGAATTTATGGTGAGCAAAGGCGA<br>AGAACTGTTTACCGGCGTGGTGCCGATTCTGGTGGAACTGGATGGCGATGTGAACG<br>GCCATAAATTTAGCGTGAGCGGCGAAGGCGAAGGCGATGCGACCTATGGCAAACT<br>GACCCTGAAATTTATTTGCACCACCGGCAAACTGCCGGTGCCGTGGCCGACCCTGG<br>TGACCACCCTGAGCCATGGCGTGCAGTGCTTTGCGCGCTATCCGGATCATATGAAA<br>CAGCATGATTTTTTTAAAAGCGCGATGCCGGAAGGCTATGTGCAGGAACGCACCAT<br>TTTTTTTAAAGATGATGGCAACTATAAAACCCGCGCGGAAGTGAAATTTGAAGGCG<br>ATACCCTGGTGAACCGCATTGAACTGAAAGGCATTGATTTTAAAGAAGATGGCAA<br>CATTCTGGGCCATAAACTGGAATATAACTTTAACAGCCATAACGTGTATATTACCG<br>CGGATAAACAGAAAAACGGCATTAAAGCGAACTTTAAAATTCGCCATAACATTGA<br>AGATGGCGGCGTGCAGCTGGCGGATCATTATCAGCAGAACACCCCGATTGGCGAT<br>GGCCCGGTGCTGCTGCCGGATAACCATTATCTGAGCACCCAGAGCAAACTGAGCA<br>AAGATCCGAACGAAAAACGCGATCATATGGTGCTGCTGGAATTTGTGACCGCGGC<br>GGGCATTACCCTGGGCATGGATGAACTGTATAAAGAATTCCATCATCATCATCATC<br>ATATGTAAT

p_EGFP_inteinN	pCMV-GFP (Addgene #11153)	ATGCATCATCATCATCATCACGTGAGCAAGGGCGAGGAGCTGTTCACCGGGGTGGT<br>GCCCATCCTGGTCGAGCTGGACGGCGACGTAAACGGCCACAAGTTCAGCGTGTCC<br>GGCGAGGGCGAGGGCGATGCCACCTACGGCAAGCTGACCCTGAAGTTCATCTGCA<br>CCACCGGCAAGCTGCCCGTGCCCTGGCCCACCCTCGTGACCACCCTGACCTACGGC<br>GTGCAGTGCTTCAGCCGCTACCCCGACCACATGAAGCAGCACGACTTCTTCAAGTC<br>CGCCATGCCCGAAGGCTACGTCCAGGAGCGCACCATCTTCTTCAAGGACGACGGC<br>AACTACAAGACCCGCGCCGAGGTGAAGTTCGAGGGCGACACCCTGGTGAACCGCA<br>TCGAGCTGAAGGGCATCGACTTCAAGGAGGACGGCAACATCCTGGGGCACAAGCT<br>GGAGTACAACTACAACAGCCACAACGTCTATATCATGGCCGACAAGCAGAAGAAC<br>GGCATCAAGGTGAACTTCAAGATCCGCCACAACATCGAGGACGGCAGCGTGCAGC<br>TCGCCGACCACTACCAGCAGAACACCCCCATCGGCGACGGCCCCGTGCTGCTGCC<br>CGACAACCACTACCTGAGCACCCAGTCCGCCCTGAGCAAAGACCCCAACGAGAAG<br>CGCGATCACATGGTCCTGCTGGAGTTCGTGACCGCCGCCGGGATCACTCTCGGCAT<br>GGACGAGCTGTACAAGAGATCTCGAGCTCAAGCTTCGAATTCTGCAGTCGACGGTA<br>CCGCGGGCGATAAAGGCATTGAATGCAGCGTGTTTAGCATTGATAGCAACGGCATT<br>GTGTATACCCAGCCGATTGCGCAGTGGCATCATCGCGGCAAACAGGAAGTGTTTGA<br>ATATTGCCTGGAAGATGGCAGCATTATTAAAGCGACCAAAGATCATAAATTTATGA<br>CCCAGGATGGCAAAATGCTGCCGATTGATGAAATTTTTGAACAGGAACTGGATCTG<br>CTGCAGGTGAAAGGCCTGCCGGAATAA

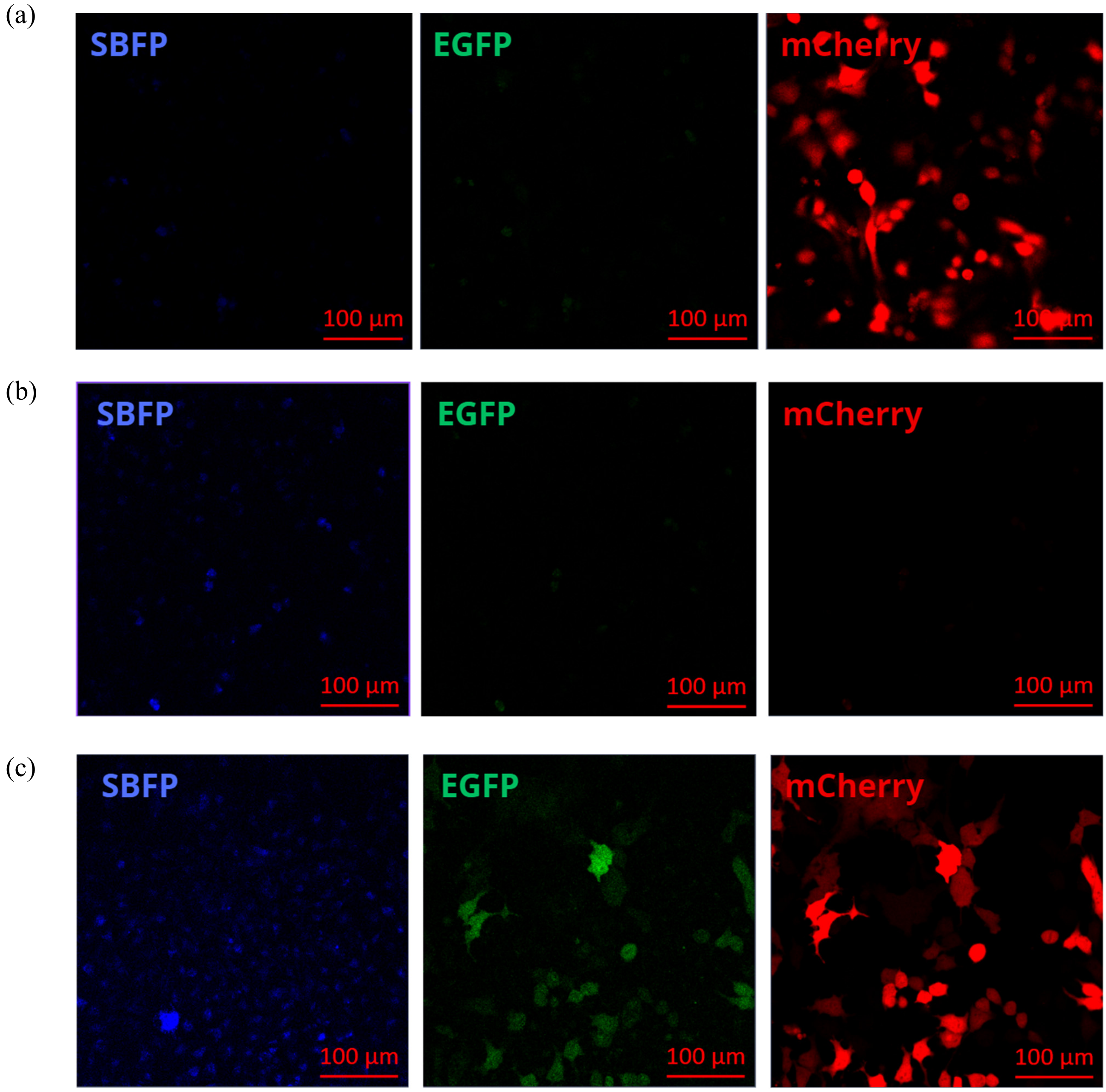

Figure S2. Representative confocal images of HeLa cells treated either with (a) p_egfp1-10_inteinN, (b) p_inteinC_egfp11, or (c) both plasmids simultaneously.

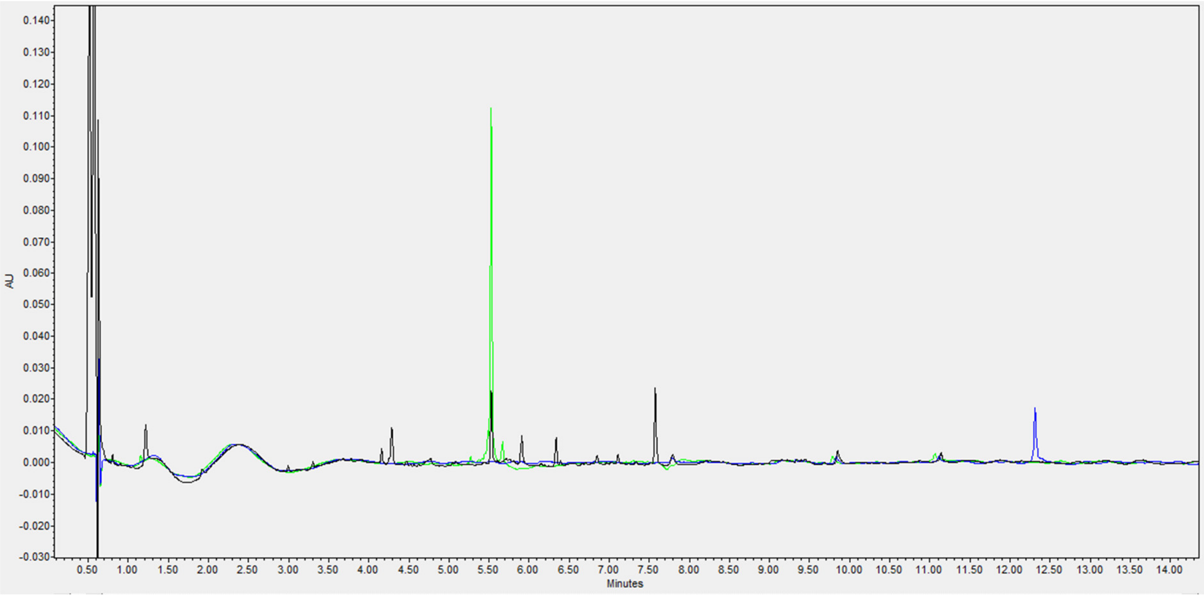

Figure S3. UPLC image. The green line is the intein^{C} peptide, the blue line is the NF55-XK(Rhod)Xpra peptide, and the black line is the purified click reaction mixture. Note the reduction in the amount of intein^{C}, which was initially in excess compared to NF55-XK(Rhod)XPra, and the disappearance of NF55-XK(Rhod)XPra from the reaction mixture. Furthermore, the emergence of a new compound (NF55-XK(Rhod)X–intein^{C} conjugate) can be observed.

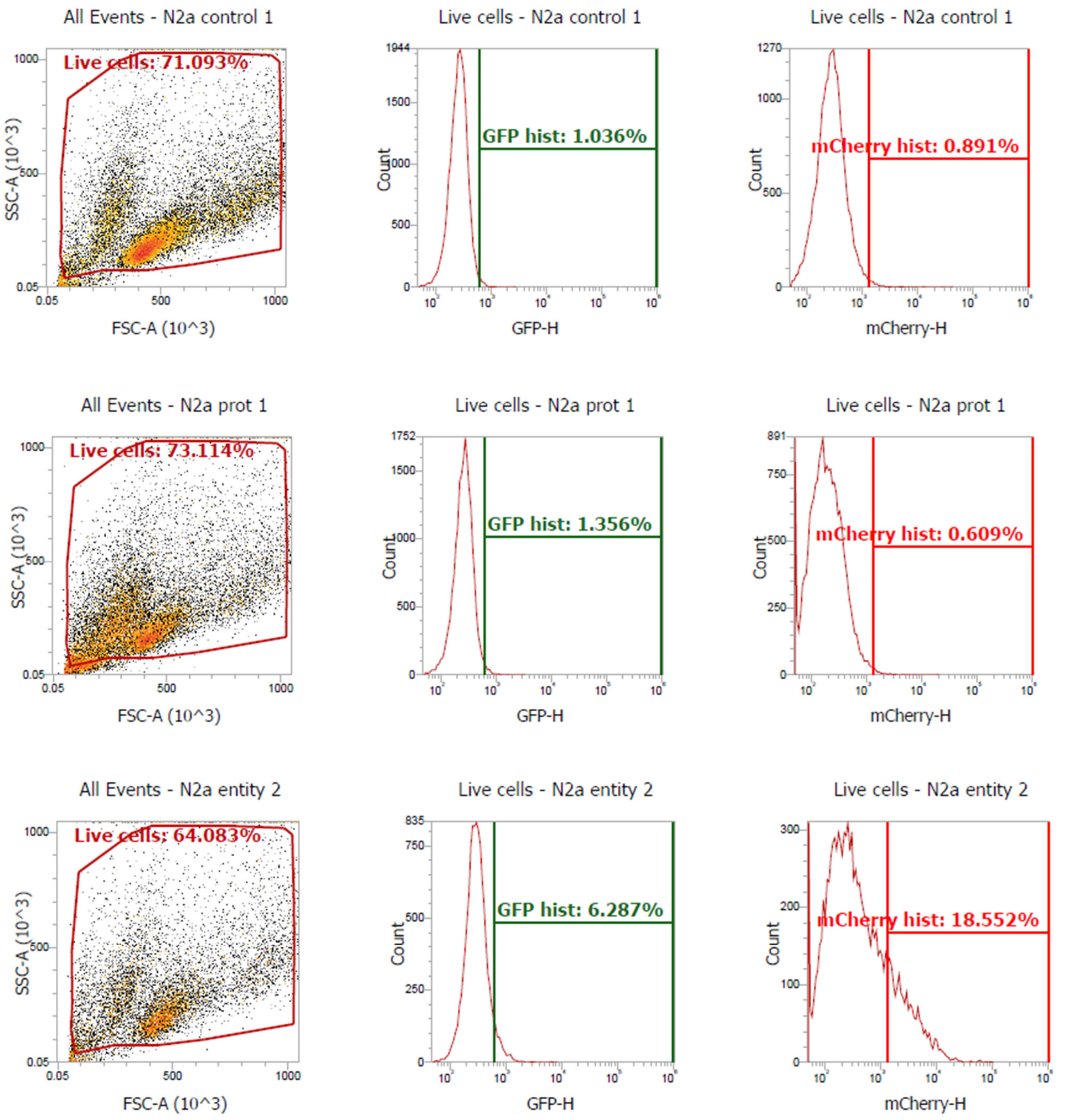

Figure S4. Representative flow cytometry plots of Neuro 2a cells transduction efficacy assay. Flow cytometry was performed using an Attune™ NxT Flow Cytometer equipped with a 488 nm argon laser. The viable cell population was determined from a plot of forward-scattered (FSC) vs. side-scattered (SSC) light. Attune™ NxT Software 3.2.1 software was used to analyze a minimum of 10,000 events per sample. The area scaling factor was 1.28, with a threshold of SSC 0.3, FSC 220, SSC 360, BL1/GFP 460, and RL1/APC/Cy5 520. GFP+ and mCherry show gated events with a signal intensity over the threshold of ~1% of untreated cells.

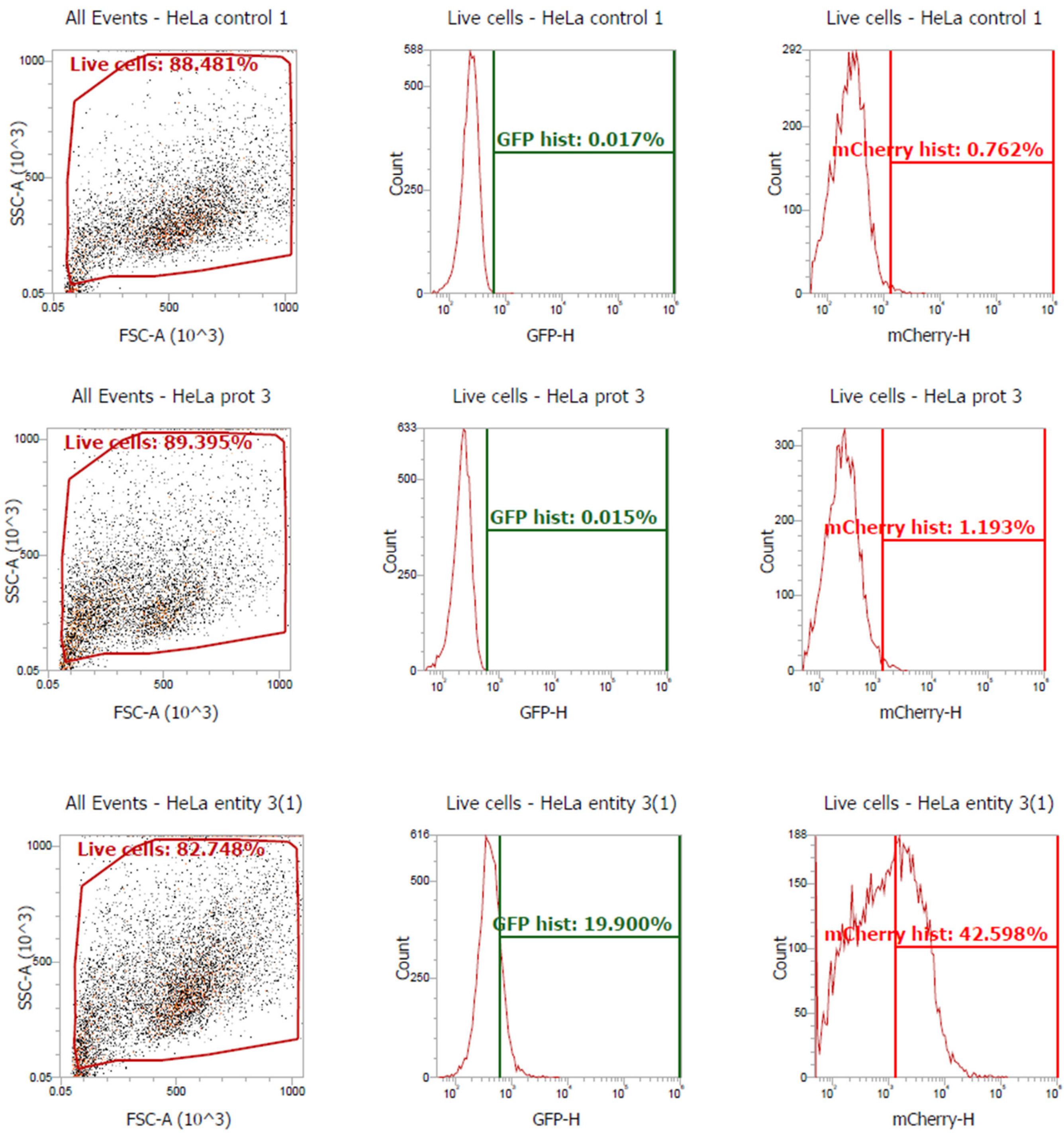

Figure S5. Representative flow cytometry plots of HeLa cells transduction efficacy assay. Flow cytometry was performed using an Attune™ NxT Flow Cytometer equipped with a 488 nm argon laser. The viable cell population was determined from a plot of forward-scattered (FSC) vs. side-scattered (SSC) light. Attune™ NxT Software 3.2.1 software was used to analyze a minimum of 10,000 events per sample. The area scaling factor was 1.28, with a threshold of SSC 0.3, FSC 220, SSC 360, BL1/GFP 460, and RL1/APC/Cy5 520. GFP+ and mCherry show gated events with signal a intensity over the threshold of ~1% of untreated cells.

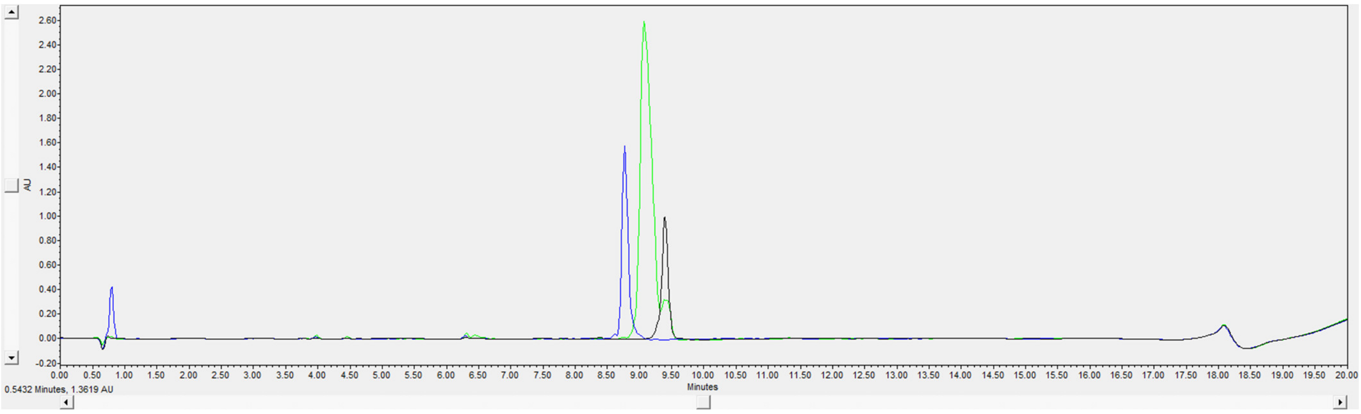

Figure S6. UPLC image. The blue line is the EGFP–intein^{N} protein, the green line is the reaction mixture of EGFP–intein^{N} protein and intein^{C}-activated NF55 peptide, and the black line is the intein^{C}-activated NF55 peptide. In the reaction mixture, no protein or peptide peaks are registered.

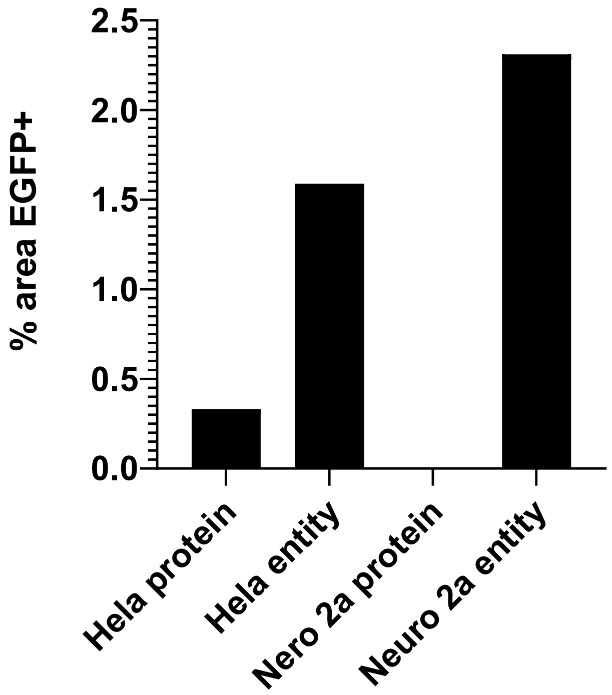

Figure S7. Confocal image analysis showing average percentage of EGFP-positive area. Analysis is performed with ImageJ (Version 1.53a), National Institute of Health, Bethesda, Maryland, USA.
